# Ear, tail and skin lesions vary according to different production flows in a farrow-to-finish pig farm

**DOI:** 10.1186/s40813-019-0126-9

**Published:** 2019-07-15

**Authors:** Alessia Diana, Laura Ann Boyle, Edgar García Manzanilla, Finola Catherine Leonard, Julia Adriana Calderón Díaz

**Affiliations:** 1 Pig Development Department, Teagasc Animal and Grassland Research and Innovation Centre, Moorepark, Fermoy, Co. Cork Ireland; 2 School of Veterinary Medicine, University College Dublin, Belfield, Dublin 4, Ireland

**Keywords:** All-in all-out, Lesions, Management, Production flow, Swine, Welfare

## Abstract

**Background:**

Pig performance and risk of disease are associated with production flow. Given the link between health and welfare, it is likely that animal welfare indicators are also associated with production flow. This study investigated the association between production flow and tail, ear and skin lesions on a farm with a purported ‘all-in/all-out’ policy. This was an observational study whereby pigs were managed according to routine farm practice. A total of 1,016 pigs born within 1 week from the same batch were followed through the production stages and the presence or absence of welfare indicators was recorded at 4, 7, 9, 12, 16 and 24 weeks of age. Three production flows were retrospectively identified: flow 1 = ‘normal’ pigs that advanced through the production stages together ‘on time’, flow 2 = pigs delayed from advancing from the 1^st^ to the 2^nd^ nursery stage by 1 week and flow 3 = pigs delayed from advancing through the production stages by > 1 week. A nested case control design was applied by matching pigs by sow parity, number of born alive and birth weight.

**Results:**

The presence of ear lesions was 4.5 less likely in pigs in flow 2 and 2.9 times less likely in pigs in flow 3 (*P* < 0.001) compared to pigs in flow 1. Pigs in flow 3 were 2.2 more likely to have tail and 1.6 times more likely to have ear lesions (P < 0.001) compared to pigs in flow 2. Pigs in flow 2 were less likely to have tail lesions compared with pigs in flow 1 (*P* < 0.05). Differences between production flows for the risk of skin lesions varied according to age (P < 0.05).

**Conclusion:**

All production flows were associated with a high risk of lesions which raises concerns for pig welfare. However, risks for ear, tail and skin lesions varied according to each production flow likely due to the specific management practices inherent to each flow. Results from this study could be used to modify existing management practices, thus leading to improvements in animal welfare and possibly performance in intensive pig systems.

## Background

All-In/All-Out (AIAO) is a management strategy that has several advantages for pig production such as improved biosecurity, health and growth performance [1, 2]. In a true AIAO system pigs are closely matched by age and they move forward through the production stages in the same groups, i.e. in the same production flow, with no re-mixing and no exposure to pigs of different ages [2]. When a group of pigs moves on to the next production stage the rooms they leave are completely emptied, cleaned and disinfected. Ultimately, a farm that follows a strict AIAO policy should minimize disease transmission [1, 2]. However, strict adherence to AIAO is difficult in practice as it is influenced by the quality of management on the farm, the level of staff training on the principles of AIAO, disease patterns/outbreaks, economics and the farm layout, among others. Hence, in reality while farmers may proclaim to follow the principles of AIAO they often inadvertently fail to adhere to them. For example, in a recent survey of 79 Irish pig farms, a high proportion of farmers claimed to practice strict AIAO in the nursery (87.3%) and finisher (84.8%) stages. However, on 33.3% of the farms declaring to practice AIAO older pigs were mixed with younger pigs in the nursery stage and on 20% of the farms declaring to practice AIAO this happened in the finisher stage (unpublished data).

One of the major constraints in adhering to AIAO management in farrow-to-finish farms is the lack of facilities to exclusively house slow-growing and/or sick pigs that are removed from the “normal” production flow (i.e. ‘pull outs’). The practice of re-grading pens by size/BW on transfer to the next production stage is also widely practised [3] in an effort to achieve uniformity in slaughter weight as producers must adhere to specific BW range specifications at the time of slaughter [4]. This usually means that faster growing pigs continue to the next production stage ‘on time’ and represent the ‘normal’ flow but slow growing and/or sick pigs are delayed from moving to the next production stage, sometimes for several weeks, and are re-grouped with similar sized, though younger pigs, from the following batch. This practice increases the chances of disease transmission between different age groups and could have an adverse effect on pig performance [3]. We reported that pigs repeatedly delayed from the normal production flow were, on average, 10 kg lighter at slaughter and at higher risk of diseases such as pleurisy and pericarditis [3] when compared with pigs that followed the normal production flow although it was not possible to deduce whether these outcomes in delayed pigs were causative or explanatory.

Nonetheless, given the link between poor health and poor welfare [5] and the fact that the practice of delaying pig is associated with re-mixing [3], it is likely that delayed pigs are also at greater risk of experiencing poor welfare. Re-mixing leads to aggression as pigs fight to establish a new dominance hierarchy [6, 7] which increases stress levels [8]. Stress in turn, can trigger the performance of damaging behaviours such as ear and tail biting [9, 10] and the resulting lesions. These lesions are highly prevalent in pig production systems, for instance, van Staaveren et al. [11] showed that pigs were affected by tail, ear and skin lesions on all farms surveyed during the grow-finisher period of a cross-sectional study involving 31 Irish farrow-to-finish farms. Specifically, the authors found tail and ear lesions as the most prevalent welfare outcomes recorded in each production stage with 2.8 and 7.6% (first weaner stage), 5.9 and 9.1% (second weaner stage) and 10.5 and 3.3% (finisher stage) of pigs affected per farm, respectively.

Damaging behaviours are a serious problem in intensive pig production systems [12]; they are both a cause of poor welfare in the receiver and reflect poor welfare in the performer [9]. While a clear aetiology has not been confirmed yet, damaging behaviours are clearly a multifactorial issue and major risk factors appear to include a barren and/or a highly stocked environment [9, 13], mixing of unfamiliar animals and disruption of the dominance hierarchy [14]. The physical damage (i.e. lesions) inflicted through such behaviours not only leads to serious effects on pig performance (e.g. carcass condemnation at slaughter because of infection in the spine [15, 16]) and economic losses for farmers [17], but also contributes to a decrease in both mental and physiological welfare of the animals [18] with further consequences for their efficiency. This highlights the importance of more research on such welfare issues.

To our knowledge, there are no studies exploring the possible association between production flow and welfare indicators under commercial conditions. Therefore, the objective of this study was to expand our previously reported results [3] by investigating the possible associations between production flow and the most prevalent welfare indicators (tail, ear and skin lesions) in grow-finisher pigs in a farrow-to-finish commercial farm.

## Methods

### Animal housing and management

The study was conducted on a 1,500 sow farrow-to-finish commercial farm with a batch farrowing system of c. 80 sows farrowing per week. This was an observational study whereby pigs were managed as per usual practice on the farm and the weekly movement of animals was tracked. This farm purported to follow an AIAO policy with pigs spending 8 weeks in the nursery stage after weaning (4 weeks in the first and 4 weeks in the second nursery stages), 4 weeks in the growing stage and 8 weeks in the finisher stage. Pigs (*n* = 1,016) born within 1 week were individually ear-tagged at birth and tracked through the production stages until slaughter. Piglets were tooth clipped and tail-docked within 24 h after farrowing. Males were not castrated according to normal practice on Irish pig farms. Sow parity, number of piglets born alive and sex were recorded. Pigs were weaned at approximately 28 days of age, managed as per usual practice on the farm and the weekly movement of animals was tracked. At weaning, entire litters were moved together into the first nursery stage and housed in groups of 55 pigs (minimum space per pig = 0.30 m^2^) composed of c. 4 to 5 litters. On transfer to the second nursery stage, groups were split and mixed by size/BW in groups of 36 pigs with a minimum 0.55 m^2^ per pig. Finally, pigs were transferred to the finishing stage and housed in groups of 35 with a minimum 0.65 m^2^ per pig. Pieces of wood approximately 1 m long provided at floor level, rubber toys (i.e. a *star* shape toy with 12 protruding rubber legs provided at floor level; [EasyFix™ Rubber Products, Ballinasloe, Co. Galway, Ireland]) and/or chains hanging from the pen walls were provided as environmental enrichment.

Housing design (i.e. penning arrangement, floor type and ventilation system) was the same within each production stage. The nursery facilities consisted of 11 rooms with 16 pens each. Within each room, 8 pens were positioned to either side with a centrally positioned corridor separating them. Nursery pens had fully slatted plastic floors with a wet/dry probe feeder with eight available spaces and an automatic temperature control system with fans in the ceiling. The grower facilities consisted of 7 rooms with 16 pens each. Similar to the nursery facilities, 8 pens were positioned to either side with a centrally positioned corridor separating them. Grower pens had fully slatted concrete floors and a wet/dry probe feeder with eight available spaces was located on one side of the pen. An automatic temperature control system with fans in the ceiling was used. Finally, the finisher facilities consisted of 38 individual trobridge houses with fully slatted concrete floors and natural over pressure ventilation. In each trobridge house with a wet/dry probe feeder with eight available spaces was located on one side of the pen. In all stages, pigs had access to water via at least one nipple drinker in each pen and wet-feed was provided ad libitum for nursery [18.3% crude protein (CP) and 10.5 MJ/DE per kg of feed]; grower (18.1% CP and 10.0 MJ/DE per kg of feed), and finisher (16.9% CP and 9.9 MJ/DE per kg of feed) pigs.

Mortality was recorded during the trial. Eight-hundred-and-twenty-four pigs reached slaughter age. All animals were slaughtered within 1 week, regardless of their body weight, at 24 weeks of age and were retrospectively classified into three production flows according to the time they spent in each production stage [i.e. flow 1 = normal (*n* = 620 pigs), flow 2 = delayed by 1 week (*n* = 111 pigs) and flow 3 = delayed by > 1 week (*n* = 93)].

### Welfare measurements

At weaning, 7, 9, 12, 16 and 24 weeks of age, pigs were individually inspected for the presence of **ear** (superficial bites but no blood; or evidence of bites/teeth marks with fresh blood and/or infection; or partial or total loss of the ear [19]); **tail** (i.e. evidence of chewing or puncture wounds, but no evidence of swelling; or evidence of chewing with swelling and signs of possible infection; or evidence of chewing with severe swelling/infection or an open wound where the tail used to be [17]), and **skin** lesions arising from aggression (several superficial scratches not penetrating the full dermal thickness; or deep cuts / lesions with or without red/dark scabs or severe laceration with infected wounds and/or dark scabs [20]) by a single trained observer.

### Data management and statistical analysis

Sow parity, number of piglets born alive and birth weight are associated with growth performance traits [21]. Thus, ANOVA tests were conducted on data from all 824 animals that reached slaughter to assess where differences in these parameters were present between production flows. Statistical differences were observed between flows for these three variables (for more information in Calderón Díaz et al. [3]). Therefore, a nested case control design was applied, whereby pigs from the three production flows were matched by sow parity, number of piglets born alive and birth weight. The matching process yielded a final data set including 120 pigs in flow 1, 60 pigs in flow 2 and 60 pigs in flow 3.

Tail, ear and skin lesions were only observed in 7, 1 and 1 pigs at weaning, respectively; thus this time point (i.e. 4 weeks of age) was not used in the analysis. Similarly, skin lesions were not observed in any pig at 7 weeks of age and ear lesions were only observed in 9 pigs prior to slaughter; therefore these time points were not analysed for these traits. Data were analysed using binomial logistic regression in PROC GLIMMIX of SAS v9.4 (SAS Inst. Inc., Cary, NC). Models included production flow, age of pigs when welfare indicators were recorded and their interaction as predictor variables and pig as a random effect. Alpha level for determination of significance was 0.05 and from 0.05 to 0.10 for trends. Results are presented as odds ratios (OR) with the associated 95% confidence interval.

## Results

### Animal management

**Fig. 1:**
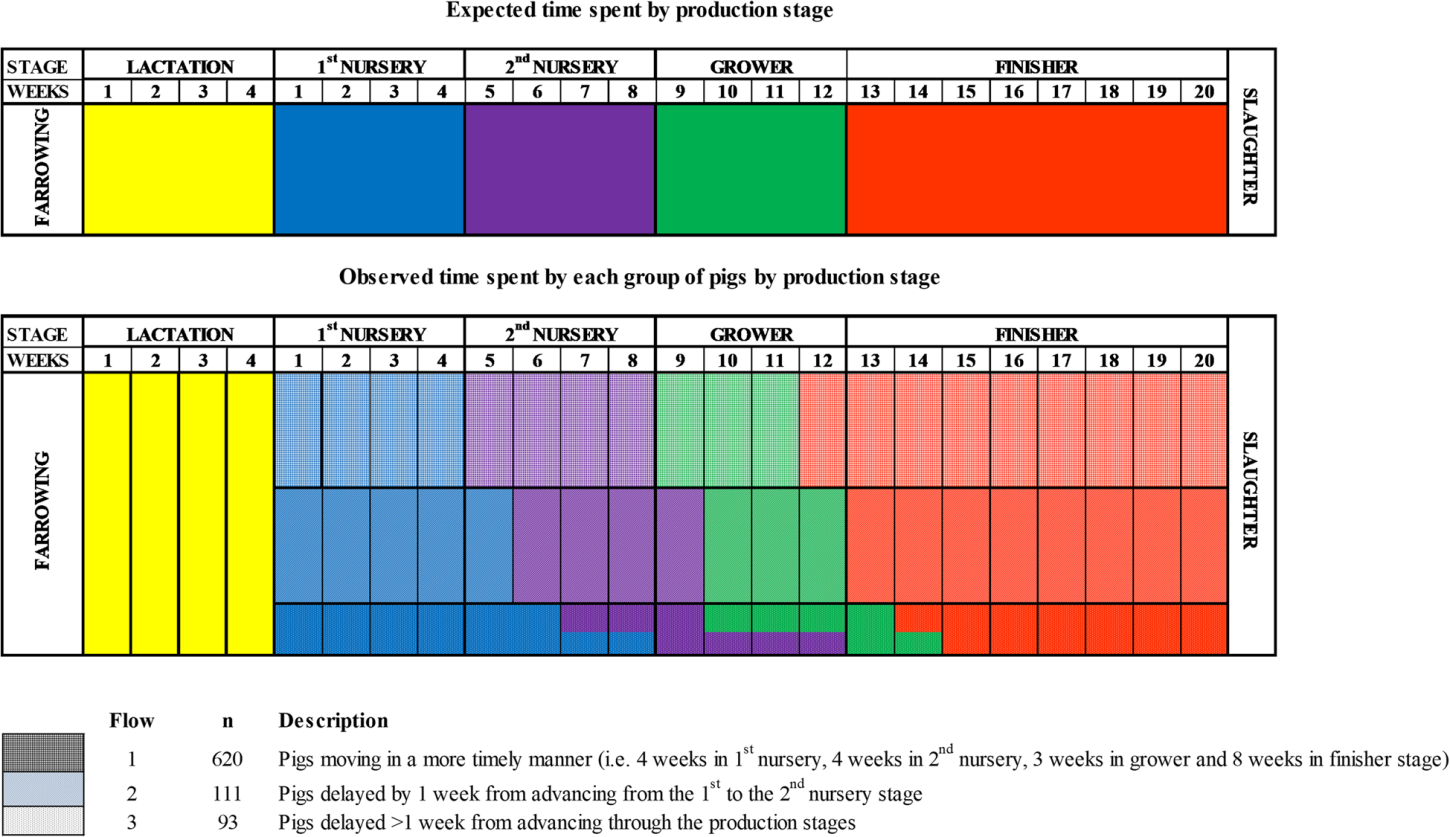
Expected versus observed time spent by each production flow in each production stage in a farrow-to-finish commercial pig farm where a batch of 1,016 pigs were followed from birth to slaughter. All animals were slaughtered within 1 week at 24 weeks of age and were retrospectively classified into three production flows (i.e. Flow 1 = ‘on time/normal’, Flow 2 = delayed 1 week and Flow 3 = delayed > 1 week) according to the time required to be moved to the next production stage

Contrary to the purported AIAO policy followed in the farm, three production flows were identified according to the time pigs spent in each of the production stages (Fig. 1).

Three weeks post weaning, the heaviest pigs in each pen were removed and re-mixed into new groups in a new room in the first nursery stage (Flow 1 pigs). Smaller pigs stayed behind in their same pen and with no unfamiliar pigs added. Pigs in flow 1 stayed in the new room in the first nursery stage for an additional week after which time they were transferred to the second nursery stage in the same groups. Pigs in flow 1 continued to move through the different production stages in the same groups and spent 4 weeks in the second nursery stage, 3 weeks in the grower stage and 9 weeks in the finisher stage. At 5 weeks post-weaning, the smaller pigs that had remained in the first nursery stage were re-graded by size/BW and the heaviest of those pigs (i.e. flow 2 pigs) were transferred to the second nursery stage while the smaller pigs were delayed once again in the first nursery stage accommodation (i.e. flow 3 pigs). Pigs in flow 2 continued to move through the production stages in the same groups and spent 4 weeks in the second nursery stage, 3 weeks in the grower stage and 8 weeks in the finisher stage. Pigs in flow 3 were mixed with younger, similar sized pigs from the following batch and with pigs that had returned from the hospital facilities having recovered from illness and/or injury. Several of the latter pigs were subsequently delayed again. It took 8 weeks to move all pigs in flow 3 to the second nursery stage, 6 weeks to move all pigs from the second nursery to the grower stage and 5 weeks to move all pigs from the grower to the finisher stage.

### Mortality and welfare indicators

A total of 18.9% of pigs died during the study. Specifically, 104 pigs died in the farrowing house (i.e. pre-weaning) which represented 54.2% of all deaths, 24 pigs died during the first and second nursery stages (12.5%), 3 pigs died during growing (1.5%), 14 pigs (7.3%) died during the finishing stage and 47 (24.5%) pigs were euthanized. These animals were selected for euthanasia on the basis of showing external abscesses and/or pathologies such as hernias, severe tail biting (i.e. complete tail loss), severe lameness or emaciation.

**Fig. 2:**
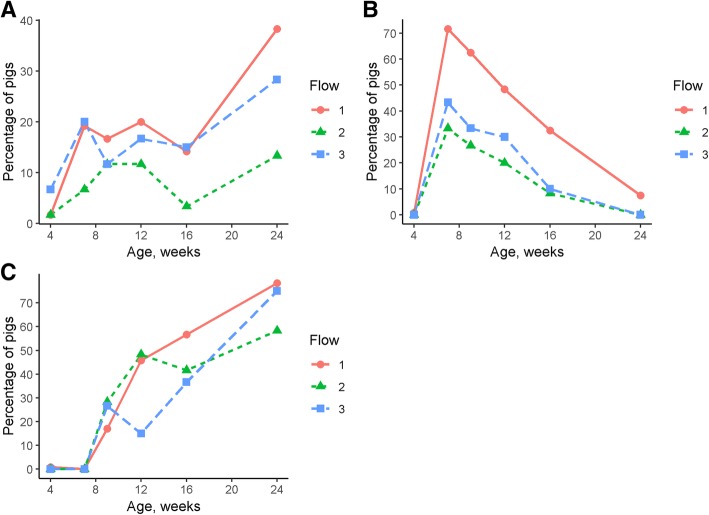
Proportion (%) of pigs affected by **a** tail^**1**^, **b** ear^**2**^ and **c** skin lesions^**3**^ at 4, 7, 9, 16 and 24 weeks of age in a group of 240 finisher pigs from one batch born within 1 week that was followed from birth to slaughter in a farrow-to-finish commercial farm. All animals were slaughtered at 24 weeks of age and were retrospectively classified into three production flows (i.e. Flow 1 = 'on time/normal', Flow 2 = delayed 1 week and Flow 3 = delayed > 1 week) according to the time they required to be moved to the next production stage. Pigs were selected from each flow in a nested case control study matched by sow parity, number of piglets born alive per litter and birth weight. ^**1**^ Evidence of chewing or puncture wounds, but no evidence of swelling; or evidence of chewing with swelling and signs of possible infection; or evidence of chewing with severe swelling/infection or an open wound where the tail used to be (Harley et al., 2012). ^**2**^ Superficial bites but no blood; or evidence of bites/teeth marks with fresh blood and/or infection; or partial or total loss of the ear (Diana et al., 2017). ^**3**^ Lesions arising from aggression and scored as several superficial scratches not penetrating the full dermal thickness; or deep cuts lesions with or without red/dark scabs or severe laceration with infected wounds and/or dark scabs (O’Driscoll et al., 2013)

**Fig. 3:**
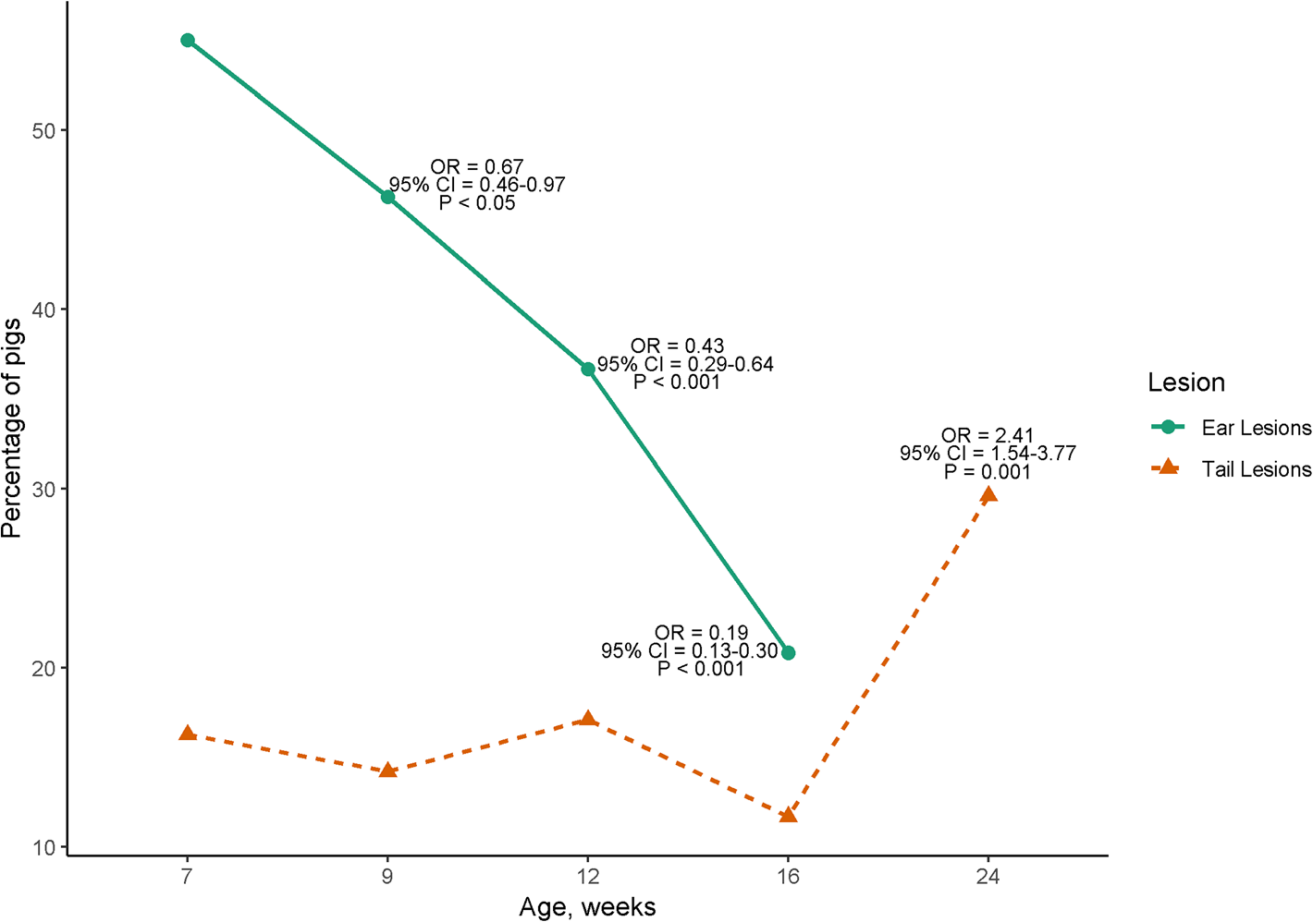
Percentage of pigs and the odds ratios (OR) ± 95% confidence interval (CI) for the presence of ear and tail lesions in grow-finisher pigs at different ages during the production cycle. Odds ratios are reported in reference to 7 weeks of age. This figure includes 240 grow-finisher pigs from one batch born within 1 week that was followed from birth to slaughter in a farrow-to-finish commercial farm

Tail, ear and skin lesions were observed in all production flows. Under the nested case-control design, there was a wide range in the proportion of pigs showing tail, ear and skin lesions during the grow-finisher period (Fig. 2). There was no interaction between production flow and pig age for the likelihood of ear lesions (*P* > 0.05). Pigs in flow 2 (OR = 0.2; 95% CI = 0.15–0.31) and flow 3 (OR = 0.3; 95% CI = 0.24–0.49) were less likely to have ear lesions compared with pigs in flow 1 (*P* < 0.05). However, pigs in flow 3 were more likely to have ear lesions compared with pigs in flow 2 (OR = 1.6; 95% CI = 1.02–2.41; *P* < 0.05). Ear lesions were less likely at 9, 12 and 16 weeks of age compared with 7 weeks of age (*P* < 0.05; Fig. 3). Similar to ear lesions, there was no interaction between production flow and pig age for the likelihood of tail lesions (*P* > 0.05). Pigs in flow 2 were less likely to have tail lesions compared with pigs in flow 1 (OR = 0.4; 95% CI = 0.25–0.60; *P* < 0.05) and pigs in flow 3 were more likely to have tail lesions compared with pigs in flow 2 (OR = 2.2; 95% CI = 1.36–3.69; *P* < 0.05). There was no difference in the likelihood of tail lesions between pigs in flow 1 and flow 3 (*P* > 0.05). There were no observed differences (*P* > 0.05) in the likelihood of tail lesions at 9, 12 and 16 weeks of age compared with 7 weeks of age; however, tail lesions were 2.4 times more likely at 24 weeks of age when compared with 7 weeks of age (*P* < 0.05; Fig. 3).

**Fig. 4:**
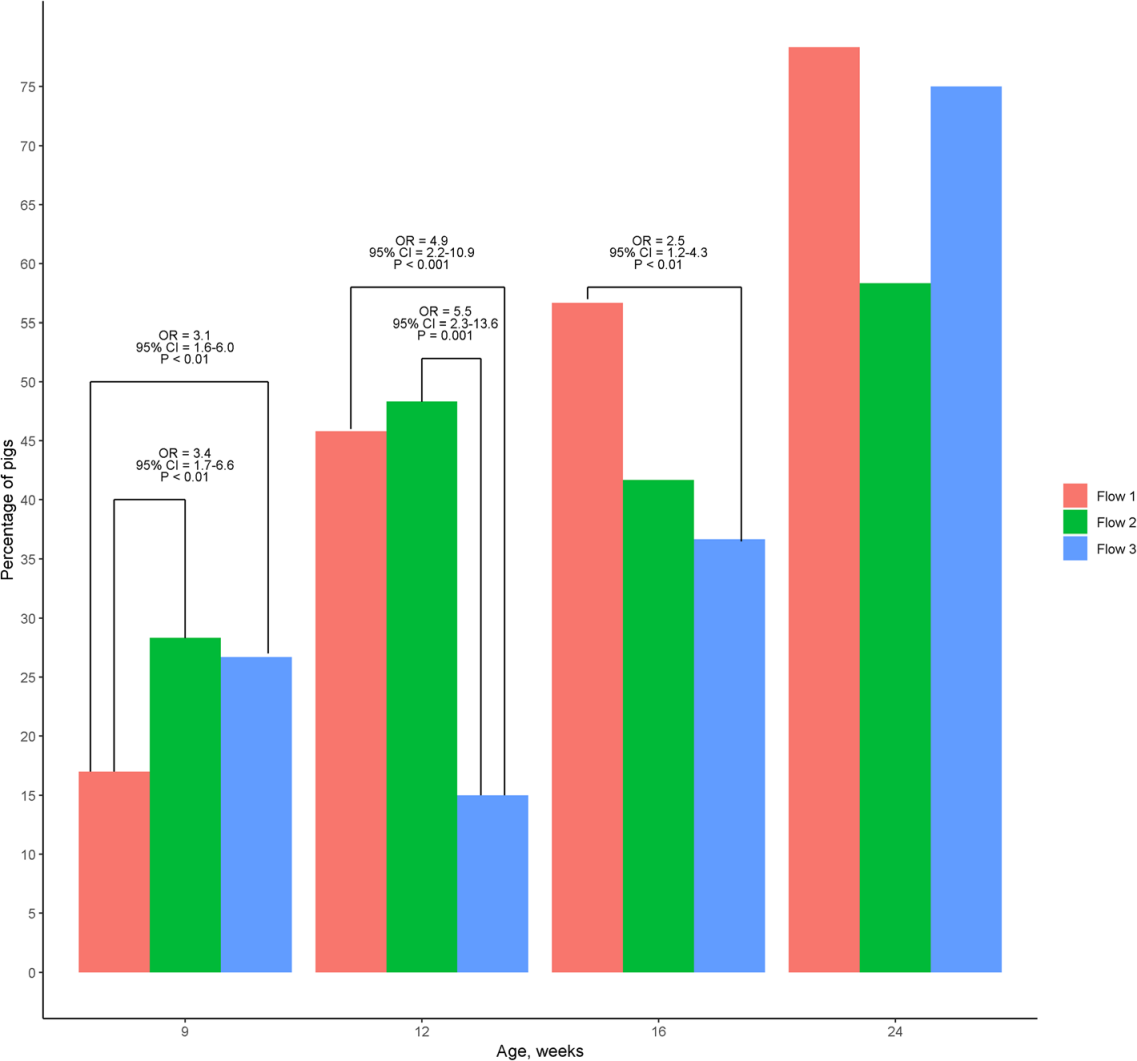
Percentage of pigs and the odds ratios (OR) ± 95% confidence interval (CI) for the presence of skin lesions in grow-finisher pigs following three production flows at different ages during the production cycle. This figure includes 240 grow-finisher pigs from one batch born within 1 week that was followed from birth to slaughter in a farrow-to-finish commercial farm. All animals were slaughtered at 24 weeks of age and retrospectively classified into three production flows (i.e. Flow 1 = ‘on-time/normal’, Flow 2 = delayed 1 week and Flow 3 = delayed > 1 week) according to the time they required to be moved to the next production stage. Pigs were selected from each flow in a nested case control study matched by sow parity, number of piglets born alive per litter and birth weight

There was an interaction between production flow and pig age for skin lesions (*P* < 0.01). At 9 weeks of age, pigs in flow 2 and in flow 3 were more likely to have skin lesions compared with pigs in flow 1 (*P* < 0.01; Fig. 4) and there was no difference in the likelihood of skin lesions between pigs in flow 2 and flow 3 (*P* > 0.05). At 12 weeks of age, pigs in flow 3 were less likely to have skin lesions compared with pigs in flow 1 and flow 2 (*P* < 0.01; Fig. 4) and there was no difference in the likelihood of skin lesions between pigs in flow1 and flow 2 (*P* > 0.05). At 16 weeks of age, pigs in flow 3 continued to have a lower likelihood of skin lesions compared with pigs in flow 1 (*P* < 0.05; Fig. 4). By 24 weeks of age, there was no difference in the likelihood of skin lesions between the three production flows (*P* < 0.05).

## Discussion

None of the flows followed the AIAO timeline declared by the farmer in each production stage. Furthermore, it was regular farm management practice to re-grade pigs according to size/BW on transfer between each of the production stages. Re-grading pigs by BW/size is a common practice in pig farms whereby producers try to minimise BW variation at the time of slaughter as abattoirs prefer more uniform batches [4, 22]. However, re-grading and therefore re-grouping, is associated with stress in pigs [23]. Previous data collected at slaughter from the same batch of pigs showed an association between production flow and health and performance [3], with repeatedly delayed pigs being 10 kg lighter and at higher risk of disease at slaughter. Given this observed association and the link between poor health and poor welfare [5], we were interested in investigating whether there was an association between production flow and welfare indicators measured on the same animals throughout the production cycle.

Our results confirmed associations between welfare indicators and the production flows identified in this farm. However, this association was not as straightforward as the association between production flow, health and growth performance previously reported for these pigs [3]. In our previous work, pigs in flow 3 were associated with a higher risk of disease and poorer performance (reflected in lower carcass weights at slaughter). In the current study, both pigs that moved through the production stages on a more timely manner (i.e. flow 1) and delayed pigs (i.e. flow 2 and flow 3) were at high risk of welfare lesions although the nature and strength of the associations varied with each production flow. Overall ear, tail and skin lesions were more likely in pigs in flow 1 than in pigs in flows 2 and 3 suggesting that good health and high performance are not necessarily synonymous with good welfare as assessed using ear, tail and skin lesions. This highlights the complex and multifactorial nature of animal welfare [24–26]. Strategies such as tail-docking, applied to prevent/avoid the occurrence of tail lesion are not adequate to tackle this issue [27, 28]. In fact, as seen in other studies [11, 17], even though pigs were tail-docked, a high percentage of tail lesions was found. However, due to the observational nature of this study, further research is necessary to investigate whether such associations are causative or explanatory.

Several risk factors including high stocking densities, mixing of pigs and a barren environment may contribute to the development of damaging behaviours and their associated lesions [9, 13, 14]. This is because of their association with stress which may render pigs unable to cope with the environment [29, 30]. Pigs in flow 1 were re-graded and re-mixed into new pens in a different house in the first nursery stage 3 weeks post-weaning. This meant that they were subjected to mixing stress and to another change in their environment at a younger age than pigs in flow 2 and flow 3, and only 3 weeks after they had already experienced the numerous stressors associated with weaning [31, 32]. This could explain the higher prevalence of ear and tail lesions in pigs in flow 1.

Although pigs in flow 2 had a similar growth rate to pigs in flow 1 [3] these animals were delayed in moving from the first to the second nursery stage by 1 week and they did not experience re-mixing at 3 weeks of age. This meant that the composition of their groups did not change until they were 9 weeks of age. Notwithstanding the potential stressful effect of a diminishing space allowance as pigs grow, there are welfare benefits to keeping pigs in a stable social group [33]. This may explain the lower likelihood of ear and tail lesions in flow 2 compared to flow 1 and 3 pigs. Finally, as pigs in flow 3 were repeatedly delayed or were those that having recovered, had returned from the hospital pens, it is likely that they experienced several re-mixings. The associated aggression and stress would have increased their risk of incurring welfare related lesions.

In this study, there was an interaction between production flow and pig age for the likelihood of observing skin lesions. Pigs perform aggressive behaviour either when they have to establish a new hierarchy due to change of group composition [34] or when there is increased competition for access to important resources. Fast growing intensively produced pigs are highly motivated to eat [19, 35]. The higher likelihood of skin lesions in flow 2 and flow 3 pigs compared to flow 1 pigs at 9 weeks of age can be explained by their recent re-mixing as this corresponds to being moved to the second nursery stage. Flow 1 pigs had already undergone a change in their group composition 2 weeks earlier (i.e. at 7 weeks of age) and thereafter remained in the same group during the subsequent production stages. On the other hand, the greater likelihood of skin lesions in flow 1 pigs compared to flow 3 pigs during subsequent time points (i.e. at 12 and 16 weeks of age) is more likely due to greater competition for access feed. However, behaviour observations were not part of this study. Therefore, we cannot confirm this theory.

Similar to other studies [11, 36], there was a reduced likelihood of pigs being affected by ear lesions and an increased likelihood of pigs being affected by tail lesions as time progressed. Ear and tail lesions are multifactorial in nature and it is possible that they share similar risk factors [37]. However, it is also possible that different combinations of risk factors only pose a risk for ear and/or tail lesions at certain time points. Calderón Díaz et al. [37] speculated that as pigs get older they are more capable of defending their ears from attention by others such that biting pigs switch their attention towards the more easily accessible tail.

Some practical implications arising from our results are to 1) pay more attention to the requirements of fast growing pigs (e.g. feeder/space allowance, as reduced space may become a risk factor for tail biting), given the plans to more strictly implement the existing ban on routine tail docking in the EU (e.g. need for pigs with intact tails) [38]; 2) Applying an ‘all-forward’ but not an ‘all fast-forward’ management system whereby no pig is left behind from stage to stage but rather pigs are split marketed at the point of slaughter and not progressed too quickly through the production stages where their age appropriate needs cannot be met by the housing environment.

Due to the observational nature of this study, further research is necessary to establish whether the associations recorded between welfare indicators and production flow are causative or explanatory.

## Conclusion

Lesions indicative of poor welfare were present in all three production flows. The relative risk of such lesions differed between production flows and was likely associated with the challenges inherent to the different management strategies employed for each flow. Besides the obvious concerns for animal welfare these findings raise, they could also represent production inefficiencies and economic losses for pig producers. Thus, results from this study could be used as a starting point for new research to establish whether the associations between welfare indicators and production flow are causative or explanatory. While, on the other hand, to study alternative management practices that would contribute to reduce welfare issues in pig production systems.

## Data Availability

The datasets used for the results presented in this study are available from the corresponding author upon reasonable request.
